# Secure Communications for Resource-Constrained IoT Devices †

**DOI:** 10.3390/s20133637

**Published:** 2020-06-29

**Authors:** Abd-Elhamid M. Taha, Abdulmonem M. Rashwan, Hossam S. Hassanein

**Affiliations:** 1 Electric Engineering Department, Alfaisal University, PO Box 50927, Riyadh 11533, Saudi Arabia; 2 School of Computing, Queen’s University, Kingston, ON K7L 2N6, Canada; arashwan@cs.queensu.ca (A.M.R.); hossam@cs.queensu.ca (H.S.H.)

**Keywords:** IoT Security, resource-aiding, resource-lending, mobile computing security, next-generation Internet security

## Abstract

The importance of securing communications on the Internet of Things (IoT) cannot be overstated. This is especially the case in light of the increasing proliferation of IoT devices and instances, as well as the growing dependence on their usage. Meanwhile, there have recently been mounting concerns over a wide array of vulnerabilities in IoT communications. The objective of this work is to address constraints in IoT devices that are “resource-constrained”, which are devices that are limited in terms of computing, energy, communication, or range capabilities, whether in terms of nominal or temporal limitations. Specifically, we propose a framework for resource-aiding constrained devices to facilitate secure communication. Without loss of generalization, the framework’s viability is illustrated by focusing on a group of security functions that utilize message authentication codes, which is a strongly representative example of resource-intensive security functions. Aspects of the framework are further demonstrated in processing cores commonly used in commercial IoT devices.

## 1. Introduction

The Internet has long outgrown being a convenient mode of communication. Beyond its importance for myriad financial and social interactions, the Internet plays an increasingly critical role across a wide range of applications including content distribution, distributed (and/or) cloud computing, softwarization/virtualization, decentralized ledgering, and sensing and actuation. In response to these and other applications, the requirements for reliable and scalable performances have substantially evolved, along with an inevitable emphasis on communication security [1].

In the context of the Internet of Things (IoT), a “Thing” can be a sensor node or an actuation module. The communication mode for such physical things is often characterized as being machine type communication (MTC), with direct (non-centralized) interaction sometimes labeled as device-to-device (D2D) communication. A “Thing” can also be a software instance, whether logical or virtualized, e.g., a mobile agent, a crawler, or even a basic thread. Such heterogeneity in definition further necessitates the need for reliable and scalable performance. More critically, however, it challenges the realization of secure IoT communication [2,3].

Things vary in both their nominal and operational resources, i.e., resource availability or accessibility during the time. Furthermore, this heterogeneity is further complicated by aspects such as hardware design, firmware design, firmware updates, communication protocols, and communications capability. Application considerations such as mobility and traffic patterns can also vary [4,5].

### 1.1. Motivation

Various innovations have been made to realize adaptive secure communication. These innovations, however, do not apply to all device types. For example, devices such as sensors or Radio-Frequncy Identification (RFID) tags may not have suitable computational resources to handle adaptive implementation or to integrate a group of security measures that can meet enough security levels for communication purposes. Meanwhile, even with adaptability, basic asymmetry in the availability of security functions at the ends may render any secure communication unachievable. The same outcome is unavoidable if security functions at the ends are not supported by the intermediate network.

Measures are therefore needed in which resource-constrained IoT devices can overcome their inherent limitations. One manner in which this can be achieved is through collaborative security, e.g., two or more devices of similar capabilities with distributed functions of interest among themselves, including security functionalities. This “distribution” or “federation”, however, is subject to the nature of the intended functions and the degree to which they lend themselves to decentralization. Meanwhile, such solutions invariably burden the already resource-constrained nodes with further processing and communication overhead that impacts their overall lifespan.

A preferable alternative to decentralized shared functions is by engaging a proxy to security functionality. In such a setting, the resource-constrained device would seek aide from a more capable device (aider). Such aiding can be either physical (i.e., a tangible and dedicated device) or logical (i.e., a service provided through a near-access-level interface). As will be discussed in Section 2, while various forms of aiding have been discussed in the context of security, none of the aiding forms is sufficiently adaptive or comprehensive to address IoT’s heterogeneity or scale requirements.

The objective of this work is to address this void. Specifically, we introduce the architectural concepts of a general-purpose framework for secure communications by resource-constrained IoT devices and instances. Toward this, we employ notions of resource-aiding that accommodate a wide range of heterogeneities in IoT. The use of such notions is motivated by the increasing computing and communications capabilities of supporting devices, as well as by the viability of support modes previously inaccessible (e.g., edge- and cloud-computing).

### 1.2. Contributions

In prior work [6], we described the architectural considerations for resource-aided security in IoT. This work is expanded on here by proposing the architecture, offering more extensive modeling, and providing a thorough performance evaluation. Specifically, our contributions to this work are as follows:

- We directly and holistically address the void of securing heterogeneous, resource-constrained IoT devices;
- We introduce a novel adaptive framework to secure communications in resource-constrained IoT devices;
- The framework is general-purpose and accommodates the heterogeneity of devices and instances in IoT, and that of network support to security functions; and
- Viability of implementation is demonstrated through an evaluation based on commonly available processing cores.

### 1.3. Paper Organization

The remainder of this paper is organized as follows. Section 2 briefly covers the past efforts in security aiding and possible design characteristics for having an aiding approach to complement a communication entrustment framework. It also outlines different resource-aiding approaches that can be used in helping to secure next-generation network communications. Section 3 introduces the proposed general-purpose framework for securing resource-constrained IoT devices. In doing so, it motivates its requirements and highlights the framework’s main elements. Section 4 focuses on the evaluation of the resource-lending aiding approach as an integral part of the discussed resource-aiding framework. As a way of demonstration, the evaluation focuses on the use of algorithms processing message authentication codes, which are representative of the resource-intensive function for which resource-constrained devices may require support. Additional open issues are discussed in Section 5. A summary of the paper with possibilities of future work are provided in Section 6.

## 2. A Review of Resource-Aiding Modes

In this section, we discuss resource-aiding modes (or possibilities) that can be utilized in a secure communication framework. Before doing so, however, we provide a review of the relevant literature.

Existing security solutions are characteristically resource-intensive, especially those relying on cryptographic measures. This characteristic imposes a significant load on computational resources of the communicating entities, in turn leading to noticeable performance degradation for secured communications. This has motivated recent research and commercial efforts to provide resource-aiding solutions that offer prompt and efficient resource-availability to secure device communications.

Examples of these efforts include secure sockets layer/transport layer security (SSL/TLS) acceleration [7], application delivery controllers (ADCs) [8], hardware-accelerated cryptography [9,10], and the security as a service (SECaaS) model [11,12]. Many of these solutions have proven to be effective in their targeted areas in providing enhanced and secured communications. However, most of these solutions were designed with technology-specific insights and with little consideration for the possibility of inter-communications between networks of different technologies and infrastructures.

Research in the IoT and other areas (e.g., information-centric Networks (ICNs)) have shown considerable advances in the development of mobile and location-independent entities [13]. Similarly, advances in the areas of software-defined radios (SDR) [14], software-defined networks (SDNs) [15], and SDN protocols, such as OpenFlow [16], have facilitated flexible and cost-effective development and productions process of a wide array of devices, as well as their supporting access and core networks. Such advances, however, introduced complexities in managing secure communications, especially for the exponentially increasing number of devices in IoT [5]. They also challenge the capabilities of existing security frameworks.

There is an evident increasing demand in realizing a generalized, high-level network abstraction that facilitates inter-device communications while allowing for a wide variety of resource capabilities. This demand can be observed, for example, in the introduction of protocols and standards for ICN and IoT based on older technologies that are used in existing Internet infrastructure. Examples of the new standards include HTTP/2 [17], constrained application protocol (CoAP) [18], which is based on the representational state transfer (REST) model used by older standards such as HTTP, and the reliance of some proposed content delivery protocols for ICN to deliver content via an existing protocol such as HTTP and RTP/RTCP [19].

There are two specific heterogeneities characteristic of future communications: entity and infrastructure heterogeneity. For entity heterogeneity, we note that future entities may include all identifiable and communicable objects (e.g., web services, a sensor, a self-publishing content, a mobile device). Normally, those entities have various characteristics and communication protocols and cannot communicate without a mediator layer or device. Meanwhile, certain entities may not have their own physical resources and will require a physical host from where they can communicate (e.g., virtualized instances) [20].

As for infrastructure heterogeneity, communicating entities may have similar processing capabilities and use the same software-level communication protocols. However, the underlying physical infrastructure of such devices may be different [21] (e.g., one end may rely on IPv6 while the other on 6LoWPAN) [22]. To ensure seamless communication between entities of different infrastructures, communication mediators must be incorporated within the required physical interfaces to allow inter-communication between the involved infrastructures.

This work is concerned with generating a high-level abstraction with an emphasis on resource-aiding. This facilitates accommodating device heterogeneity in terms of resources. In the following sections, we review possible security resource-aiding modes. We also identify the key factors involved in the design and implementation of an adaptive security resource-aiding platform.

### 2.1. Security Gateways

Security gateways are dedicated entities equipped with multiple interfaces that provide secured communication-relay services between entities and networks of different characteristics and requirements [23]. Examples include virtual private network (VPN) gateways, firewalls, HTTP/TLS/SSL proxies, and IoT/ICN network gateways. Such gateways typically provide three common services: address translation, interface translation, and enhanced services.

(1) Address translation: In some address-limited networks, such as private IP networks, entities are considered internal and cannot be addressed directly from outside. Gateways act as address translators that help in relaying communication between internal entities and external networks.
(2) Interface translation: Specialized networks such as IoT or cellular usually have different low-level communication protocols than IP networks. Gateways in such networks have protocol translation engines, allowing seamless and abstracted communication between applications from different network technologies. Such interferences thus lessen the need to interface or application redesign.
(3) Enhanced aervices: Service providers and communication entities may demand certain security level requirements, e.g., having a minimum-security strength or a digital signature. They may also have requirements for communication performance levels, e.g., a minimum latency. Gateways usually apply enhanced services through revising and applying security measures to the information relayed between entities to meet the security and performance demands. The revision procedure can take one of two forms: either changing the relayed messages themselves on behalf of the sending entities or and offering post-receiving services. Verification of the received data is an example of the latter.

There are two key advantages to using gateways as communication security aiders. First, they provide privacy and security through filtering malicious messages, limiting inbound access, and unmasking the true identities of communicating entities. Secondly, they use gateways aids scalability and applicability. Specifically, gateways can be designed to be scalable in terms of the services offered and the type of networks serviced. They can also eliminate the need to redesign communicating entities from networks utilizing different technologies.

Such advantages have made security gateways a popular security aiding approach in the industry, especially as they facilitate secure inter-entity communications while avoiding the need for major infrastructure and/or entity overhaul. Despite this, however, security gateways have limitations. The use of security gateways, for example, does not allow the communicating entities any control over how their messages can be altered as they pass through the gateways. A vulnerable gateway also results in vulnerable communication for the entity.

The design complexity of a security gateway is also a potential drawback, especially with the existence of various network technologies to translate between, and in the absence of a high-level application communication protocol. Gateways are also commonly designed with translation interfaces that target the specific networks they interconnect, e.g., LoRa and IP networks. Introducing redundancy in the gateway design may also unviable as serviced networks may not offer support for multiple interfacing or entity migration. Only one coordinator per network, for instance, is supported in ZigBee (IEEE 802. 15. 4) network.

### 2.2. Resource-Lending (or Offloading) Engines

Resource-lending or offloading enables a communicating entity to access and utilize the resource(s) of an external entity to process messages or computations [24]. While gateway processes deliver on behalf of the communicating entity, a resource-lending gateway returns the processed message to the entity so that the latter can proceed with the transmission.

**Figure 1:**
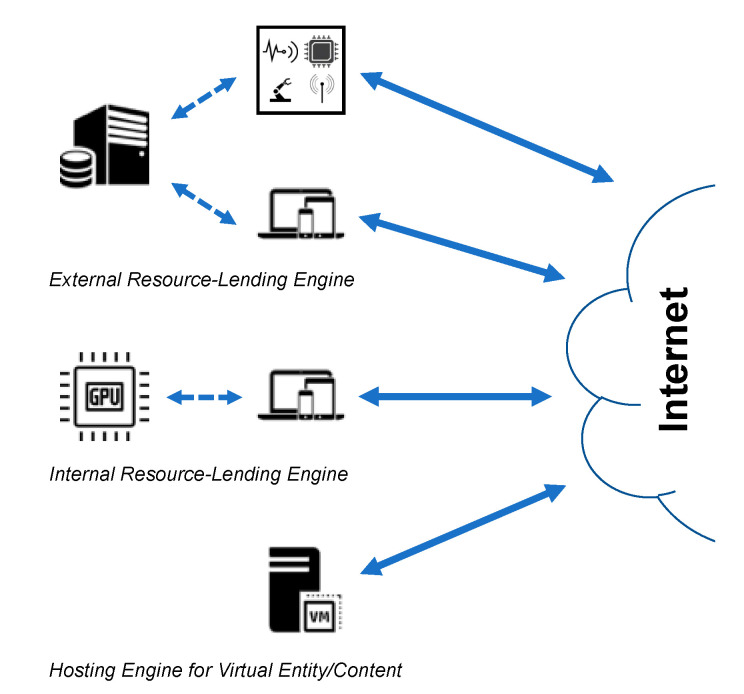
Different classes of resource-lending setups.

There are three classes of resource-lending engines that depend largely on how the interaction is managed between the engine and the communicating entity. These classes are illustrated in Figure 1 and can be described as follows.

(1) Internal lending engines: Also known as hardware accelerators, internal lending engines are a type of coprocessing hardware integrated into entities to aid in the efficient handling of complex operations such as processing graphics and cryptography [25]. Existing implementation examples include cryptographic coprocessors [9] and Transmission Control Protocol (TCP) accelerators [26].

Since hardware accelerations are integrated within their serving entities and are usually protected from external direct access, they are not subject to the same message communication threat that exists with non-secured gateways. However, hardware accelerators can be compromised if their hosting entities are compromised, a vulnerability usually made possible through an exploit that allows direct unrestricted access to the entity’s memory and input/output (I/O).

Hardware accelerators are an alternative to software-only solutions as they provide a cost-effective energy-efficient means of high-performance computing for complex specialized tasks. However, they still demand energy from their hosting entities especially if the accelerator is used for general-purpose computations, e.g., graphics processors [27]. Moreover, resource-constrained entities, such as battery-operated sensors, may choose not to integrate complex security hardware accelerators due to cost and energy constraints. This leaves such entities with limited and less-scalable security services when compared to the use of external aiding approaches such as gateways and external offload engines.

(2) External lending engines: Like gateways, external lending engines are also specialized communicating entities that provide communication services. They differ from gateways in that they work like the internal hardware accelerators. They are therefore logically separate from their serviced communicating entities and do not forward communications on behalf of their serviced entities.

External lending engines and gateways share the advantage of achieving scalable and flexible setup. However, each approach has a disadvantage. Gateways take message processing control away from communicating entities. If a gateway is compromised, then any passed communications are also compromised. With external lending engines, communicating entities have full control of their messages and can switch or suspend using offload engines if they are found to be compromised. Meanwhile, external lending engines do not have any control over how entities communicate with other ends, which leads to their inability to protect communicating entities from external attacks. Besides, using external lending engines usually results in additional communication overhead for the communicating entities due to the piggybacking of messages between the lending engine and the serviced entity.

(3) Hosting engines: The next generation of virtual entities, such as multimedia content and portable services, can be uniquely identifiable and mobile. They naturally cannot communicate, however, without being hosted on a physical resource such as a content server or a virtual processing server. In this view, a hosting engine is essentially a physical resource that accommodates virtual entities and communicates on their behalf. Hosting engines are further responsible for providing communication identification and security services for the hosted virtual entities. This is regardless of whether they are affiliated with the hosted entities, e.g., as in the case of virtual networks and ICNs [28].

Since hosting engines aim for virtual entities, any capable physical communicating entity can act as a hosting engine as opposed to having dedicated aiding approaches in the case of external lending engines and gateways. This viability, however, imposes a challenge in implementing consistently secured entity identification especially in the absence of standardized virtual communication identification. In addition, all virtual entities share the strengths and weaknesses of their hosting engines. When a host is compromised, all its hosted entities are also compromised. Network access control in such cases can be challenging since there is the possibility of virtual entities migrating from their compromised host to another.

### 2.3. Witness (or Guarantors)

A witness or guarantor mediates between a sender and receiver to monitor and validate exchanged communication between the pair. The validation is performed in a non-involved manner [29]. In other words, no processing or communication is delegated to the witnesses to be performed on behalf of the communicating entities.

Scenarios, where witnesses are engaged, are ones where the communicating entities retain full control of the session with the witness provides some level of security. The process requires setting up additional channels (sender-witness, witness-receiver) over which the witness provides its service to the endpoints.

For example, a witness can offer integrity measures if unavailable at the endpoints. Alternatively, witnesses can authenticate either the communicating entities and/or their communication state. In such instances, if the original session is rerouted by an attack, the witness can identify inconsistencies in the original session, and communicate these inconsistencies to the sender. Further applications of witnesses are illustrated in [30].

Notwithstanding, the general appeal of witnesses as aider is to provide the minimal (or “better-than-nothing”) security support to resource-constrained entities. Such a solution can readily be observed to be limited to specific applications. It can also be observed to introduce substantial vulnerabilities if adopted in a generalized manner in IoT.

## 3. A Framework for Adaptive Secure Communications in IoT

This section introduces our adaptive secure communication framework. In doing so, we highlight the design requirements needed to realize aiding for resource-constrained devices. Meanwhile, support for heterogeneity is realized by allowing for different aiding possibilities. An overview is also provided for the critical elements of aiding resource discovery, operation, and risk management.

### 3.1. Design Requirements

A successful framework for resource-aiding is inevitably dependent on three main aspects: accommodating entity heterogeneity, secured access to entities, and establishing trustfulness of the resource-aiding operation.

In what follows, we elaborate on each of these elements.

(1) Accommodating entity heterogeneity: We take an expanded view of entity mobility, accommodating both physical and virtual (logical) communications. This readily entails the need for different services and their varying expectations. Since it is a challenge to have one design that accommodates all, a prospective security resource-aiding framework should incorporate modularity and abstraction within its design to allow for easy integration with various applications and communication technologies. Any security resource-aider under such a framework should be able to easily (and, where applicable, promptly) integrate modules for the services they provide. Such aiders should further benefit from the secured standardized service announcement interfacing that offers services and resource capabilities for the interested entities.
(2) Secured access to entities: A security resource-aider cannot provide services to the requesting entities if the aider cannot gain access to the entities that it intends to serve. Restricted access can be due to risk management actions, e.g., malware quarantining, or prevention measures, e.g., firewall access restrictions. For a prospective security resource-aiding framework to be successful, access restrictions should be considered in its design. This means that the next-generation of security resource-aiders may need to seek and obtain, if applicable, permission to operate unrestrictedly within the networks they intend to serve. These aiders may also need to provide a minimum level of security guarantees to be able to successfully obtain such permission. Therefore, these aiders must be designed with measures to ensure protection from possible attacks and uncontrolled tampering to their services and network access.
(3) Establishing trustfulness to the aiding operation: When an entity does not have enough computational or energy resources to execute an intensive task, an external resource-aiding service may be sought to help with that task. In many scenarios, the requesting entity may entrust the task/information to an external aider lacking enough security measures to determine if that aider is trustworthy or compromised/malicious. Any prospective security resource-aiding framework proposal should consider incorporating tools for the communicating entities to seek the trustfulness of their prospective resource-aiders. Such tools may include a trusted third-party aiding certification, an authorized third-party blacklisting, or a community-based reputation system.

By adhering to the design criteria and design directions, it is possible to achieve a scalable and adaptive resource-aiding framework for communication security. The detailed design of such a framework is not within the scope of this document and is left for future investigations.

### 3.2. Framework Overview

For the framework to be generally applicable, the type of resource-aiding applied (i.e., gateway, external lending engine, or hosting engine) should be appropriated to the nature of the communicating entity.

- If the communicating entity is limited in its resource but requires communication with other entities that are under a different infrastructure/network, aiding gateways can be applied;
- If the entities are unable (whether nominally or due to depleting resources) to handle processing security functions, it can delegate the processing to external lending engines; and
- If the entity is virtual, a hosting engine can be used to handle communication and security service on the entity’s behalf.

Note that in the latter case, the hosting engine can itself rely on gateways or resource-lending engine for its resources, especially in cases where virtual entities are migrated between different networks.

**Figure 2:**
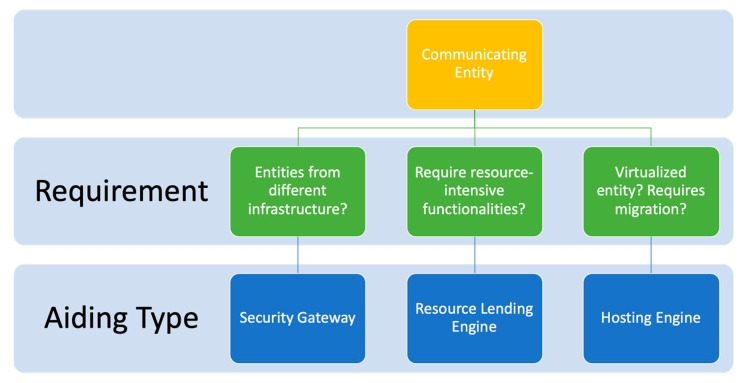
Overview of possibilities for secure communication within the framework.

The above considerations are illustrated in Figure 2. To achieve adaptive resource-aiding security services, we propose that all aiders utilize a form of adaptive communication security strategy. This flexibility maximizes the availability and efficiency of both communication security and resource utilization. As an example, we focus on the next section on analyzing the performance of external lending engines using an authentication-trim strategy.

### 3.3. Considerations for Service Discovery

For aiding-gateways, the network operator is expected to provide an updated list of fixed-location resource-aiding-gateways, essentially eliminating the need to implement a dedicated service/resource discovery mechanism. In the case of external-lending and hosting engines, however, a form of service discovery is required.

To contain vulnerabilities, the introduction of a resource-aider into a network is expected to proceed in a supervised manner that can be either centralized or distributed. Once introduced, the resource-aider resources can be communicated to the entities within the network or discoverable via a query, depending on the mode of service discovery applied.

If an entity requires additional resources to process a specific security service used in a communication session, it seeks out available resource-aiders that are capable of processing such a service. We believe that the discovery mechanism should be decentralized, i.e., using a peer-to-peer (P2P) discovery protocol, but with some centralized oversight, i.e., using blacklisting/reviewing services, like the election of aiders. Having a decentralized discovery can help efficiently maintain the discovery process in real-time for scenarios where aiders have high mobility and/or the network topology is continuously changing [31]. Meanwhile, in centralized discovery, it is up to the seeking entity to filter the discovery result list of capable aiders based on the centralized trustfulness check.

A typical resource discovery would proceed as follows. Once a filtered list of capable and available resource-aiders is populated (i.e., a resource-aiding pool), the communicating entity begins by requesting service. This resource query can be made, for example, in a round-robin manner. If a resource-aider responds to the request, the entity is linked to the aider until either the request is fulfilled; the entity migrates, or the aiding resource becomes unavailable. In the case of the latter, the entity may proceed to repeat the process. Through all, if the resource-aiding pool does not satisfy the entity’s requirement, it may proceed to revise the requested resource or revoke the request.

### 3.4. Resource-Aiding Operation

**Figure 3:**
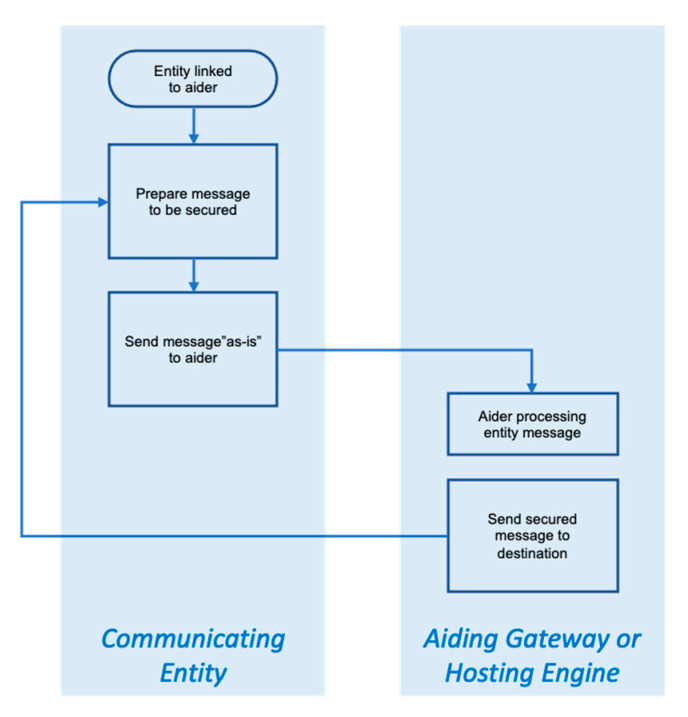
Overview of secure resource-aiding operation in the case of aiding gateway or hosting engines. In the case of the latter, the communicating entity would be within the hosting engine.

**Figure 4:**
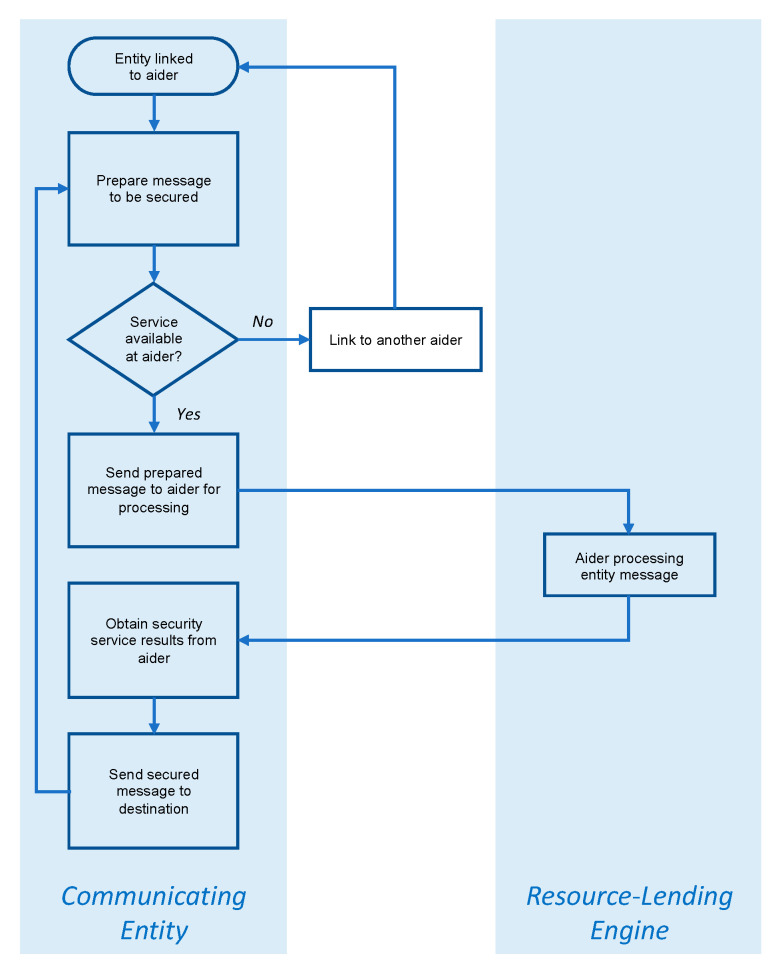
Overview of secure resource-aiding operation in the case of a resource-lending engine.

When the communicating entity is linked to the aiding resource, the actions that follow are straightforward, though naturally dependent on the nature of the aider. In the case of aiding gateway or hosting engine, the entity prepares the message to be secured and passes it on to the gateway or engine “as-is”. In turn, the gateway or engine engages the relevant security processes agreed upon, then forwards the secured message to its destination. If the entity is linked to a resource-lending engine, the engine “envelopes” the entity’s message, then pass it back to the entity for transmission. The two cases are respectively illustrated in Figure 3 and Figure 4.

It should be noted that the above does not assume a dependence on a single aider on either the sender or the destination part. For example, a virtual content entity with high access demand may need additional hosting engines to decrease the load on the originating host. Yet, another example would be when a requesting entity needs different services for different communication sessions based on the requirements and limitations of each session.

### 3.5. Managing Resource-Aiding Vulnerabilities

Entrusting foreign entities with information does not come without risk. This is true for all intermediate communication nodes in the network including gateways, router, and firewalls. However, since resource-aiders can be offered by both network operators and communities, the risk can be even higher. This is largely due to the difficulty of access-control in a distributed manner (or community-provided, federated, etc.), especially when in a network with dynamic topology due to mobility.

Therefore, to mitigate the potential risks of resource-aiding, communicating entities need to adopt certain measures, including:

- Limit the use of aiders by employing a risk-load tradeoff. Where the entity can secure its message in a resource-effective manner, it should not rely on aiders.
- Similarly, a risk-need tradeoff should be observed, whereby only the messages that require security are passed on the aider.
- Communication with the aider, and their selection, should both be conducted securely.
- The choice of aiders should be temporally varied.
- Where applicable, multiple aiders can be used for the same communication.
- For control information (i.e., processing instructions), the only necessary information should be relayed.

## 4. Performance Evaluation

In this section, we review the performance characteristics of resource-aiding approaches that can be utilized in our proposed adaptive communication security resource-aiding framework. We focus on evaluating a set of security functions, namely message authentication codes (MAC), that are used to ensure communication data integrity between entities. This choice is for demonstration and does not impact the general applicability of the framework. Meanwhile, the framework can easily be extended to other security measures with processing or latency considerations, i.e., requires the use of aiders.

### 4.1. Performance Criteria

In this study, we assume that the resource-aiders are processing MAC tags for the requesting entities using an authentication-trim adaptive strategy. Therefore, the metrics involved are:

- Total throughput. The data rate for the transmitted messages including the size of the attached MAC tags (in MB/s).
- Useful throughput. The data rate for the transmitted messages excluding the size of the attached MAC tags (in MB/s).
- Message latency. The latency of sending 1 byte to the aider for MAC processing.

### 4.2. Considerations for Evaluation

Without loss of generality, no considerations are made for keying demands or negotiations. Considerations for such granular aspects are discussed in Section 5 and will be investigated in future work. Meanwhile, the resource-aiding scenarios examined to facilitate the performance evaluation from the sending entity perspective.

Gateway and hosting engines different from lending engines in the manner in which they aid entities and may thus exhibit different profiles for processing overhead. Despite this, however, we believe that they have comparable performance characteristics. Studying the performance heterogeneity between the gateways and hosting engine is beyond the scope of this paper but will be considered in future work.

### 4.3. Evaluation Environment and Scenarios

The environment is designed to ensure that the operating system imposes minimum influence upon it. All experiments in this study were conducted under the Linux operating system environment (Ubuntu 12. 04 LTS). The chosen operating system running in Gnome desktop mode uses the “Completely Fair” scheduler for scheduling its processes.

A well-known cryptographic library Crypto++ (v.5.6.2) is used for this study as the provider for the evaluated MAC functions. This selection was motivated by the library’s popularity among academia for studying cryptographic performance and cryptanalysis [32]. It is also open-source and has cross-platform compatibility, making it suitable to run under various operating systems and computer architectures. Furthermore, the library has both hardware-assisted and software-only implementations for some functions such as AES (using x86 AES-NI extension) and SHA-256/512 (using x86 SSE-2 extension), making it a good option to test the effect of different implementations utilizing the same hardware.

To further understand resource utilization and message latency impact on aiding performance, we impose a basic adaptive control for handling authentication functions which we call the authentication-trim strategy. Specifically, the strategy works on switching between powerful authentication functions when computational resources are underutilized and weaker functions when computational resources are over-utilized. To simplify analysis and inference, we focus on two selected MAC functions for adaptation control: when the resources are underutilized, the stronger (HMAC-SHA3-512) is invoked; when overutilized, the faster but weaker (HMAC- MD5) is invoked.

To ensure the confidence level of the obtained results, each experiment in this study was run several times and could reach a steady-state before taking measurements. The communication sessions in each experiment were averaged with a 10% trimmed-mean calculation to reduce the influence of the environment context heterogeneity on unfairly affecting the throughput. Finally, an overall throughput average was calculated for all experiments conducted in correlation to the number of communication sessions.

Evaluation results were obtained for MAC functions running under the following architectures.

(1) x86-based (32-bit) laptops and tablets
  Intel Core I3 M350 (32-bit mode; Dual-core with SMT),
  Intel Core I5 650 (32-bit mode; Dual-core with SMT),
  Intel Pentium 4 M 3. 0 GHz (Single-core with SMT),
  Intel Atom D525 (Dual-core with SMT), and
  AMD Opteron 2354 (32-bit mode; Quad-core).
(2) x86-based (64-bit) laptops and tablets
  Intel Core I5 650 (64-bit mode; Dual-core with SMT).
(3) ARM-based (32-bit) smartphones and tablets
  Texas Instruments’ DM3730 ARM Cortex A8 (Single-core) and
  Texas Instruments’ OMAP 4460 ARM Dual-core Cortex A9 (Dual-core).

The above architectures are representative of both existing and future mobile systems in the market. While newer processors continue to emerge, especially with some possessing more cores and instruction extensions, operation optimization patterns and techniques did not exhibit significant changes. For example, techniques such as core-parking, frequency-stepping, hardware multi-threading, thermal throttling, etc., are still maintained. The following evaluations, therefore, still reflect and scale the expected performance characteristics for emerging processors.

Finally, two scenarios were considered:

(1) Gateways/hosting engines: Where entities forward messages to be completely handled by their aider or where entities are hosted by their aider. Both the gateway and the hosting engine are acting as a regular entity for the authentication-trim strategy.
(2) External offload engine: Where entities send messages to their aider and then obtain the corresponding MAC tags. We assume two control strategies. The first control strategy is in the hands of the requesting entity; meaning that the requesting entity maintains the authentication-trim adaptation control while obtaining the trim-tradeoff from the aider. The second control strategy is that the aider has full control of the adaptation.

### 4.4. Latency Overcoming Throughput

**Figure 5:**
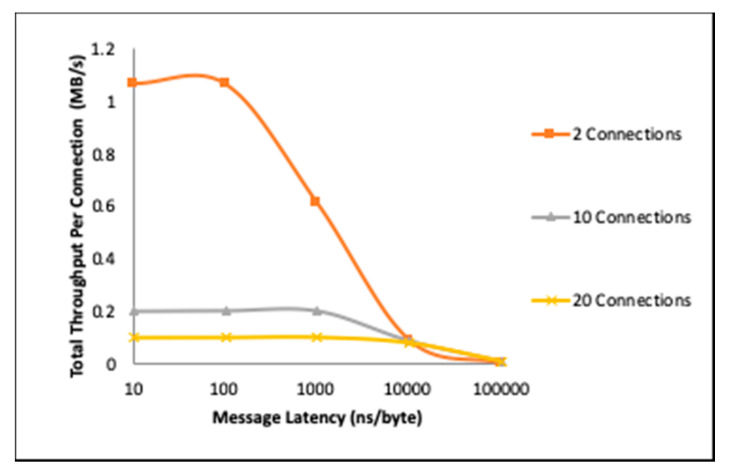
Impact of message latency on throughput at the aiding-gateway. (Tested on Texas Instruments’ DM3730 ARM Cortex™ A8, 1.0 GHz).

Figure 5 shows the total throughput of an aiding-gateway with message delivery at the rate of 100ns/byte, 1000ns/byte, and 10000ns/byte, and with the hosting engine using the same physical setup as a reference. All MAC tagging was processed with the strongest available MAC function (HMAC-SHA3-512), indicating that both the host and the gateways computational resources were not sufficiently utilized to trigger a security power down.

It is critical to note here that while the measured value in Figure 5 and the following figures is throughput, the objective is not to showcase throughput improvement by resource aiding. In Figure 5, for example, the throughput shown is per connection, and the decrease in throughput as latency increases is directly attributed to underutilization, and not to switching load, i.e., the decreased message arrival rate at the aider “frees” the processor time.

With this in mind, we can observe that as the message latency increases, the resource utilization by the aider decreases rapidly and the total throughput becomes correlated more to the message latency than to the resource demands of the MAC processing. This is most prominent in the case of two connections, where the processor throughput “peaks” at around 100ns/byte, then begins to drop as the message arrival rate becomes less than the processor service rate. For 10 and 20 connections, this “peaking” can be observed at the 1000ns/byte and 10000 latency points, respectively.

Figure 5 also shows that when message latency is too small, especially with a lower number of connections, the throughput slightly decreases. This reflects the aider’s processing resources being over-utilized, which is a critical observation. Based on this, aider adaptation can be optimized not only based on processor loading but also based on measured or expected message latency.

With the above in mind, we next proceed with examining the impact of aiding at both the gateway and the communicating entity.

### 4.5. Aiders Overcoming Message Latency

**Figure 6:**
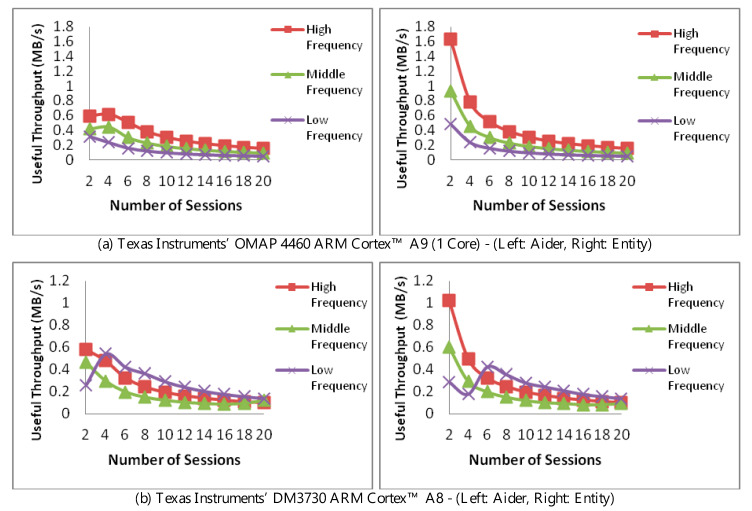
Performance of resource-aider vs. resource-equivalent entity running the authentication trim strategy at different CPU frequencies.

Figure 6 compares the impact of processing MAC functions at the resource lending aider (left, top, and bottom) and the communicating entity (right, top, and bottom). Throughput is measured against the number of communication sessions and using a single core. For both, a 100 Mbps connection is used. For the aider, however, a message latency of 1000ns/byte is used. To isolate the impact of any frequency optimization, the operating frequency was fixed at the highest, middle (median), and lowest frequencies.

In light of the above, adaptation was limited to the aforementioned authentication-trim. The result of the trim can be observed in the low frequency in the bottom two figures, where a higher throughput was achieved due to switching to a lower (lighter) MAC scheme. Due to handling the message exchange, the trim took place at four connections in the left figure, while taking place at six connections in the right.

One aspect of authentication control placement can be appreciated in comparing the two figures in Figure 6b. Here, the aider’s useful throughput (left) is less than when the authentication is performed at the entity (right). This difference is to the message latency and overhead between the aider and the entity, slowing down the performance at the aider, while directly handled at the entity, i.e., without delays due to message exchange.

For Figure 6b, the “peaking” in the low-frequency performance is due to authentication trim, allowing low-frequency processing to yield higher throughput whether at the aider or the entity, with a higher throughput achieved shortly at the entity (in the range of 4–8 connections).

There are three conclusions to be drawn here. The first is that processing delegation naturally impacts overall throughput. A decision mechanism is hence needed to decide when is it optimal to seek the aider’s resources. Secondly, the aider’s involvement must take into account its overall loading. Finally, the use of authentication trims at aiders carry may allow for overcoming throughput losses due to latency and message exchange.

### 4.6. Impact of Authentication Control Placement

**Figure 7:**
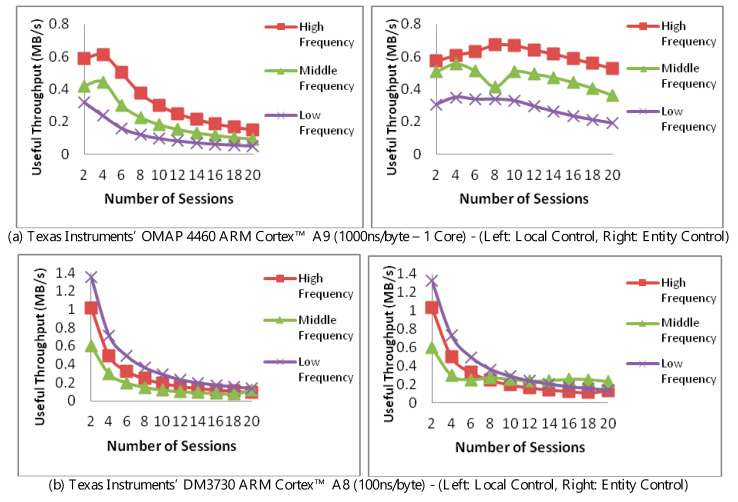
Performance of resource-aider with local and requesting entity adaptation control with varying CPU frequencies.

In Figure 7, we compare two scenarios while monitoring the throughput at the aider. In the first scenario (left, top, and bottom), the aider has full control of the adaptation decision based solely on the MAC processing performance. In the second scenario (right, top, and bottom), the requesting entity uses the message exchange latency with the aider’s trim-tradeoff to instruct the aider of the adaptation decisions. Throughput is measured against the number of communication sessions and using a single core. The message latency for the top and bottom pairs was respectively set at 1000ns/byte and 100ns/byte. As before, frequencies were fixed at high, middle, and low to isolate the impact of any frequency optimization.

An elaboration is needed on the consequences of placing the control at the entity, i.e., right, top, and bottom. To facilitate this, the aider would report its loading status to the entity which, in turn, would respond with a suggested trim, i.e., choice of authentication, if needed. In this manner, the processing of the authentication takes place at the aider, but the choice of the authentication happens at the entity.

The peaking in Figure 7a (left) is due to loading and is similar to that observed in Figure 5, i.e., not due to the authentication-trim. The extended peaking and higher throughput observed in Figure 7a (right) is due to the availability made by latency in both messages and relaying the control signals, with the fluctuation in the middle frequency at eight connections due to decision between tried authentication trims.

To distinguish the impact of control placement from the trim, we can observe the throughput in Figure 7b. In this figure, message latency is set at 100 ns/byte, i.e., higher arrival rate than that of Figure 7a. In the left figure, we note that a lower authentication is selected at the low-frequency, hence yielding a higher throughput. A similar observation can be made for low-frequency in Figure 7b, right when the number of sessions is between 2 and 10. When the number of connections is 12 and above, however, the middle frequency becomes more dominant. This change is largely due to the aider managing two things in the presence of increased arrival rate, managing the trim, and the processing the entity’s control.

Two aspects become apparent here. The first is that although aider autonomy reduces delay and messaging overhead entity control, it remains desirable in instances where the entity needs to satisfy an overall latency constraint. The second, which is more subtle, is the intricacy of establishing performance when various optimizations are not directly controlled. Care should therefore be exercised when deciding on performance figures when it comes to judging the applicability of an aiding-based security measure.

## 5. Remarks on Some Open Issues

Our focus on secure resource-aiding in this work stems from two inevitabilities: the need to avail working options in extending Internet security when and where needed, and the limitation of a substantial number of devices in IoT. As a solution, resource-aiding introduces its own set of vulnerabilities, some of which we addressed in Section 3.4. There are, however, other considerations to be made, which we discuss below.

### 5.1. From Framework to Implementation

Security resource-aiders are meant to serve entities and networks of various types and requirements. This, in turn, results in a challenge for designing a standardized resource-aiding system, as not all aiders are going to serve the same types of entities and networks. However, the security resource-aiders are not intended to be a complete solution on their own and are proposed herein as a complement to a fuller solution targeting more extensive communication and network scenarios.

To address design diversity challenges in implementing security resource-aiders, we can utilize a modular security service provider framework design. Resource-aiders need only a basic service layer and can obtain or load security service modules as demanded by the requesting entities. For addressing heterogeneity in physical layer communication demands, the resource-aiders can also incorporate SDR and SDN modules to address those demands. Such granular design would further be subject to thorough security analysis and comparison with similar solutions [33].

### 5.2. Aiding Form

References to aiders in this work have largely been made in abstraction while considering dominating aiding techniques. It should be understood, however, that an aider need not be a singular entity. The notion of federated IoT, for example, captures an operational mode that lends itself naturally to security, e.g., [34]. Through such a view, both thing/entity and aider become dually physical and logical. Similarly, the notion of edge/fog computing (i.e., in the sense of the cloud extended to the access network) [35], further expands possibilities for aiders, especially in forms of proliferated and/or systematized security services [36].

### 5.3. The (Cyber-Physical) Systems View

In evaluating the notions discussed in this work, we noted assumptions on managing security level based on the processor temporal capability. The results also indicated the interaction between message latency and aiding effectiveness. Indeed, considerations for IoT security cannot be made in isolation of other cyber or physical phenomena with which it interacts [34]. This further spans to physical consideration within the cyber modules, e.g., processor overheating or actuation failure [37]. An expanded view is thus needed in generalizing frameworks for IoT security [38].

### 5.4. Privacy

As with any system entrusted with information, there is always a privacy concern since the information is usually shared by a foreign entity. In an ideal scenario, the communicating entity should not send information for further processing without applying its security measures such as using an available weak security measure. However, if the originating entity does not have the resources or the other end does not accept the originating entity’s security measures, the entity must entrust the information to be processed completely by a security aider.

Addressing privacy with security aiders is very challenging. Unlike network-operated services, aiders are usually provided by the community. Even with trust scoring and aider certification in place, there is no guaranteed solution to ensure that aiders do not retain information sent by the requesting communicating entities. If privacy is important for a resource-constrained entity, and it cannot use its security measures, it may opt-in a partial aiding support, whereby, the transmitted information is partially secured to avoid sharing fully useful private information with the aiders.

## 6. Conclusions

In this paper, we investigated the use of security resource-aiding entities to assist in securing communications for resource-constrained IoT devices and instances. Different aiding approaches were explored, with their strengths and weakness outlined. We then introduced a holistic framework that minds device and instance heterogeneity in both nominal and operational resources. As a way of demonstration, we conducted a performance evaluation aiding in message authentication codes. We observed that when giving the adaptation control to the requesting entity, the variation in the message exchange latency is to have aggressive behavior on the adaptation control. It thus appears that adaptation control is better handled at aiders. In turn, having control at the aiders further means that instances of message latency can be exploited to increase authentication levels. The work concluded with a discussion of relevant open ends.

Based on the findings presented in this work, efforts currently underway are focusing on the design and implementation of a detailed protocol for secure resource-aiding, and we evaluated its effectiveness. The protocol will expand on the considerations presented herein to include both physical aspects of processing and exhaustive communication considerations.
